# Preparation and Evaluation of the Cytoprotective Activity of Micelles with DSPE-PEG-C60 as a Carrier Against Doxorubicin-Induced Cytotoxicity

**DOI:** 10.3389/fphar.2022.952800

**Published:** 2022-08-04

**Authors:** Beihua Xu, Zhongpeng Ding, Ying Hu, Ting Zhang, Senlin Shi, Guangmao Yu, Xuchen Qi

**Affiliations:** ^1^ College of Pharmaceutical Sciences, Zhejiang Chinese Medical University, Hangzhou, China; ^2^ School of Pharmaceutical Sciences, Zhejiang Pharmaceutical University, Ningbo, China; ^3^ Department of Thoracic Surgery, Shaoxing People’s Hospital, School of Medicine, Zhejiang University, Shaoxing, China; ^4^ Department of Neurosurgery, Sir Run Run Shaw Hospital, School of Medicine, Zhejiang University, Hangzhou, China

**Keywords:** doxorubicin, DSPE-PEG-C60, micelles, cancer, apoptosis, ROS

## Abstract

To deliver doxorubicin (DOX) with enhanced efficacy and safety *in vivo*, fullerenol-modified micelles were prepared with the amphiphilic polymer DSPE-PEG-C60 as a carrier, which was synthesized by linking C60(OH)_22_ with DSPE-PEG-NH_2_. Studies of its particle size, PDI, zeta potential, and encapsulation efficiency were performed. DOX was successfully loaded into the micelles, exhibiting a suitable particle size [97 nm, 211 nm, 260 nm, vector: DOX = 5:1, 10:1; 15:1 (W/W)], a negative zeta potential of around −30 mv, and an acceptable encapsulation efficiency [86.1, 95.4, 97.5%, vector: DOX = 5:1, 10:1; 15:1 (W/W)]. The release behaviors of DOX from DSPE-PEG-C60 micelles were consistent with the DSPE-PEG micelles, and it showed sustained release. There was lower cytotoxicity of DSPE-PEG-C60 micelles on normal cell lines (L02, H9c2, GES-1) than free DOX and DSPE-PEG micelles. We explored the protective role of DSPE-PEG-C60 on doxorubicin-induced cardiomyocyte damage in H9c2 cells, which were evaluated with a reactive oxygen species (ROS) assay kit, JC-1, and an FITC annexin V apoptosis detection kit for cellular oxidative stress, mitochondrial membrane potential, and apoptosis. The results showed that H9c2 cells exposed to DSPE-PEG-C60 micelles displayed decreased intracellular ROS, an increased ratio of red fluorescence (JC-1 aggregates) to green fluorescence (JC-1 monomers), and a lower apoptotic ratio than the control and DSPE-PEG micelle cells. In conclusion, the prepared DOX-loaded DSPE-PEG-C60 micelles have great promise for safe, effective tumor therapy.

## 1 Introduction

The use of doxorubicin (DOX), one of the anthracyclines, is limited by its serious side effects, such as cardiotoxicity and hepatotoxicity, as an anticancer drug in clinical use. Previous studies have reported that DOX exerts its toxic effects *via* a combination of reactive oxygen species (ROS)–mediated oxidative stress and lipid peroxidation, which are frequently associated with heart, liver, kidney, and lung damage (Pugazhendhi et al., 2018).

Researchers have utilized many nanoparticles to deliver DOX to enhance its antitumor activity and avoid drug accumulation in the heart. Nanoformulations, including liposomes, nanoparticles, and micelles loaded with DOX, have been tested in clinical trials and approved by the FDA for cancer treatment, such as Mycoet® and Doxil®. PEG-coated Doxil® is a so-called stealth liposome that has a much longer half-life, reduced plasma volume distribution, and low toxicity in comparison with that of free DOX (James et al., 1994).

However, clinical studies found that although PEG modification provides the benefit of low cardiotoxicity, it has the disadvantage of high skin toxicity. PEG-modified Doxil® tends to aggregate in the skin, causing “hand-foot syndrome,” characterized by numbness, paresthesia, tingling or pain, and swelling, with a 50% incidence after a course of injection treatment (Zhao et al., 2021). Doxil® also has a risk of bone marrow suppression, severe leukopenia, and even severe respiratory distress (Merle et al., 2017).

Fullerene (C60) is a cage-like spherical carbon molecule with a variety of biological activities. Polyhydroxylated fullerenes (fullerenols) have demonstrated high antioxidative activity and radical scavenging activity *in vivo* and *in vitro* (Wang et al., 1999; Chen et al., 2012; Mashino, 2022). Researchers have reported the protective effects of fullerenols toward DOX-induced cardiotoxicity and hepatotoxicity and their potential role in the protection of the lungs, kidneys, and testes in rats treated with a high dose of DOX *in vivo* (Injac et al., 2009a; Injac et al., 2009b; Borović et al., 2014; Seke et al., 2016; Petrovic et al., 2018). However, experiments demonstrated that a high dose (up to 100 mg/kg) of fullerenol may be needed to achieve the desired organ protection result in rats (Jacevic et al., 2017) due to the concentration-dependent effects of fullerenol on antioxidative viability, which indicated that high-dose treatment is required in clinical applications. Meanwhile, previous studies have reported that fullerenols have a rapid clearance speed from blood with a half-life of less than 1 h, and they are mainly metabolized by the liver and excreted through urine in mice (Song et al., 2010). Therefore, prolonging the half-life of fullerenols *in vivo* is an important way to increase their bioavailability and decrease the necessary dose.

Many articles have reported that attaching PEG to molecules or nanoparticles decreases their clearance rate from the body because PEG polymers can protect them from the recognition and phagocytosis by the reticuloendothelial system (RES) and prolong their half-life in the bloodstream (Babu et al., 2014; Rastogi et al., 2020; Rommasi and Esfandiari, 2021; Cheng et al., 2021; Kunitskaya et al., 2021).

**FIGURE 1:**
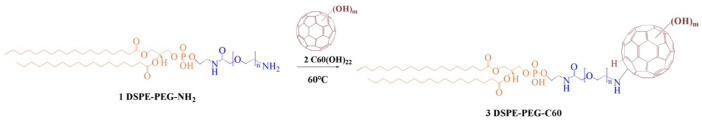
Synthesis of DSPE-PEG-C60.

The amphiphilic polymer distearoyl phosphatidylethanolamine-polyethylene glycol (DSPE-PEG) is often used in pharmaceutics as a carrier and it has a strong tendency to form micellar structures in water. In this study, a covalent combination of C60(OH)_22_ and DSPE-PEG-NH_2_ was accomplished in a one-step facile way, obtaining fullerenol-grafted distearoyl phosphatidylethanolamine-polyethylene glycol (DSPE-PEG-C60). When the molar ratio of DSPE-PEG-NH_2_ to C60(OH)_22_ was set to 1:1, product 3 was found to be the major product after 48 h of coupling (Figure 1). An improvement of DSPE-PEG-C60 circulation in the blood is expected due to the PEG protection and difficult glomerular filtration of molecular clusters used as nanopharmaceutics.

Polymeric micelles have also received great attention for tumor drug delivery, such as Genexol-PM, a clinically approved nanoformulation loaded with paclitaxel (Ahn et al., 2014), and NC-6004, a micelle loaded with cisplatin and coated with polyethylene glycol that is in preclinical trials (Subbiah et al., 2018). Polymer micelles are known for their advantages, such as increased drug solubility, long cycle effect (PEG block), and easy mass production. In this article, DOX-loaded micelles were composed of triblock copolymers, with PEG as a nonimmunogenic coat, C60(OH) as a coloaded attenuator as well as a carrier, and distearoyl phosphatidylethanolamine (DSPE) as a biodegradable core-forming agent. The *in vitro* cytotoxicity and antioxidative stress of these micelles were also studied.

## 2 Materials and Methods

### 2.1 Materials

Doxorubicin hydrochloride (DOX, also called Adriamycin, Shanghai Jizhi Biochemical Technology Co., Ltd.), fullerenol (C60(OH)_22_, Hengqiu Technology Co., Ltd.), and DSPE-PEG (1000)-NH_2_ (Yuxi Pharmaceutical Technology Co., Ltd.) were used in this study.

### 2.2 Synthesis of Fullerenol-Grafted Distearoyl Phosphatidylethanolamine-Polyethylene Glycol (DSPE-PEG-C60)

Fullerenol (C60(OH)_22_) (316 mg, 0.275 mmol) and distearoyl phosphatidylethanolamine-polyethylene glycol-amino (DSPE-PEG-NH_2_) (500 mg, 0.275 mol) were mixed together in water (10 ml). The reaction solution was stirred for 2 days at 60°C. After dialysis (MW 1000) against deionized water three times, the solution was lyophilized for two days, and a brown product (DSPE-PEG-C60) was obtained with 85% yield.

### 2.3 Characterization of DSPE-PEG-C60

#### 2.3.1 Nuclear Magnetic Resonance, Infrared Spectroscopy, and Differential Thermal Analysis

The NMR spectra were recorded on a Bruker advance 500 nuclear magnetic resonance (NMR) spectrometer using tetramethylsilane (TMS) as an internal reference. D_2_O was used as the solvent for fullerenol, DSPE-PEG-NH_2_, and DSPE-PEG-C60. The infrared (IR) spectra were recorded on a Fourier transform infrared (FTIR) spectrometer (Thermo Fisher Nicolet iS50). KBr tablets of fullerenol, DSPE-PEG-NH_2_, and DSPE-PEG-C60 were used in the IR instrument. The IR spectra were acquired after 16 scans with a scanning range of 400–4,000 cm^−1^. Differential Scanning Calorimetry (DSC) was performed using a Mettler Toledo DSC. The detection conditions were 30–300°C, the heating rate was 15°C/min, and the N_2_ flow was 100 ml/min (El-kharrag et al., 2011; El-kharrag et al., 2012; El-kharrag et al., 2019).

### 2.4 Preparation of Micelles

Solutions of DSPE-PEG-C60 (or DSPE-PEG-NH_2_) with different concentrations (10 mg/ml, 20 mg/ml, and 30 mg/ml) and a solution of DOX (2 mg/ml) in distilled water were prepared. Then, DSPE-PEG-C60 (or DSPE-PEG-NH_2_) and DOX were mixed 1:1 by volume, and the mixture was heated to 60°C and slowly stirred for 30 min. The solutions were then cooled and filtered through a membrane (0.22 μm) to obtain the DOX-loaded micelles.

### 2.5 Characterization of Micelles

#### 2.5.1 Transmission Electron Microscopy and Dynamic Light Scattering

The diameter and morphology of the DOX-loaded micelles were obtained by transmission electron microscopy (TEM, H-7650, Hitachi, Japan). The micelle solutions were dropped onto a carbon-coated copper grid and dried in air at room temperature for 5 min, and the morphology of the micelles was immediately observed with a transmission electron microscope. The size distribution, zeta potential, and PDI of the micelles were determined by dynamic light scattering (DLS) using a Zetasizer Nano ZS-90 (Malvern, United Kingdom). Data were recorded at 25°C with a scattering angle of 90° (Nazarbek et al., 2021; Xie et al., 2021).

#### 2.5.2 Determination of DOX Loading Capacity and Encapsulation Efficiency

The samples were prepared according to a previous method and then evaluated *via* ultrafiltration as reported (Meng et al., 2019). The micellar solutions were added to the upper centrifugal tube (MWCO 30,000, Millipore, Burlington, MA, United States) and centrifuged at high speed (12,000 r/min) for 20 min. The DOX concentration of the filtrate was determined by the HPLC method, obtaining the free DOX content in the micellar solution (W1). The micellar solutions were then destroyed by methanol, added by the mobile phase to a constant volume, and the DOX concentration was determined by HPLC to obtain the total DOX content (W2) in the micellar solution. The mass of the carrier in the solution is W3. The drug loading content (LC) and encapsulation efficiency (EE) were calculated by the following formulas:

EE=(W2−W1)/W2×100%,

LC=(W2−W1)/(W2+W3)×100%.

#### 2.5.3 Release Kinetic Studies of Micelles

The prepared micelle solutions (1 ml) were put into dialysis bags (MW 5000), which were soaked in phosphate buffer release medium (pH 7.4, 31 ml) in flasks. The closed flasks were incubated at 37°C with slow stirring. The released DOX concentration was measured at a predetermined time point using a UV–Vis spectrophotometer. The release rates of DOX from the micelles were determined by dividing the amount of DOX released within a certain period of time by the initial DOX content of the micelles.

### 2.6 Cell Lines and Cell Culture

Human hepatocellular carcinoma (HCC) cell lines (BEL-7402 and HepG2), human gastric carcinoma cell lines (SGC-7901), immortalized normal human hepatocytes (L02), immortalized normal human gastric mucosal epithelial cells (GES-1), and immortalized normal rat cardiomyocytes (H9c2) were obtained from the Cell Bank of the Chinese Academy of Science (Shanghai, China). H9c2, a subclone of the original clonal cell line derived from embryonic BD1X rat heart tissue, is often used in the study of myocardial diseases as cardiac myoblasts. Cell lines were cultured in DMEM or RPMI 1640 medium supplemented with 10% fetal bovine serum (FBS) and 1% penicillin/streptomycin at 37°C and 5% CO_2_.

#### 2.6.1 Cell Viability Assay

Cells were grown in 96-well plates (3×10^3^ cells/well) for 24 h and then incubated with either micelles (vector: DOX = 10:1(W/W)) or free DOX at equivalent concentrations for 72 h. Then, 10 μL of CCK-8 solution (Beyotime, CA) was added to each well. The cells were further incubated at 37°C for approximately 45 min. The absorbance was measured at 450 nm using an MK-3 microplate reader (Thermo Fisher Scientific, United States). The cell apoptosis rate was calculated according to the following formula: apoptosis rate = [(OD_450_ control well-OD_450_ administration well)/OD_450_ control well]×100%. The measurements were repeated three times, and the absorbance value of the untreated control samples was considered as 100% viability.

#### 2.6.2 Analysis Apoptosis Detection

The cellular apoptosis of micelles loaded with DOX was examined by flow cytometry. Specifically, the cells were seeded at 3 × 10^5^ cells/well density in 6-well plates for 24 h and then treated with either micelles (vector: DOX = 10:1; 15:1 (W/W)) or free DOX at equivalent concentrations of DOX for 24 h. The cells were harvested, and the FITC annexin V apoptosis detection kit containing annexin V-FITC and propidium iodide (BD Pharmingen) was used. The cells were incubated in the dark at room temperature for 15 min, measured by flow cytometry (BD, United States) and analyzed using FlowJo V10 software. The experiments were repeated three times using at least three independent biological replicates.

#### 2.6.3 Intracellular ROS Detection

ROS production upon micelle treatment was detected by 2′,7′-dichlorofluorescein diacetate (DCFDA) staining using a DCFDA-based cell kit (Biosharp). The cells were incubated in 6-well plates at 3 × 10^5^ cells/well density for 24 h and treated with either micelles (vector: DOX = 10:1; 15:1(W/W)) or free DOX at an equivalent concentration of DOX for 24 h. Then, the drug-containing medium was discarded, the cells were incubated with DMEM containing 10 μM DCFDA in the dark at 37°C for 30 min, and the cells were harvested and detected by flow cytometry.

#### 2.6.4 Mitochondria Membrane Potential Determination

The mitochondrial membrane potential was determined *via* JC-1 staining. The cells were incubated in 6-well plates (3 × 10^5^ cells/well) for 24 h and then treated with micelles (vector: DOX = 10:1; 15:1 (W/W)) or free DOX at an equivalent concentration of DOX for 24 h. Then, the treated cells were collected, stained with a JC-1 probe at a final concentration of 5 μg/ml, and incubated in the dark at 37°C for 45 min. The cells were washed with JC-1 buffer at least three times and then detected by flow cytometry in the fluorescein isothiocyanate (FITC) (for JC-1 monomers) and phycoerythrin (PE) (for JC-1 aggregates) emission ranges. The fluorometric ratio of JC-1 aggregates to JC-1 monomers was used as an indicator of mitochondrial dysfunction by assessing the changes in the mitochondrial membrane potential.

### 2.7 Statistical Analysis

All data were generated from three independent experiments and are presented as the means ± SD. The data were analyzed using Student’s t-test by Prism software version 6 (Graph Pad Software Inc., United States), and the critical level of significance was set at *p* < 0.05.

## 3 Results

### 3.1 Preparation and Characterization of DSPE-PEG-C60

**FIGURE 2:**
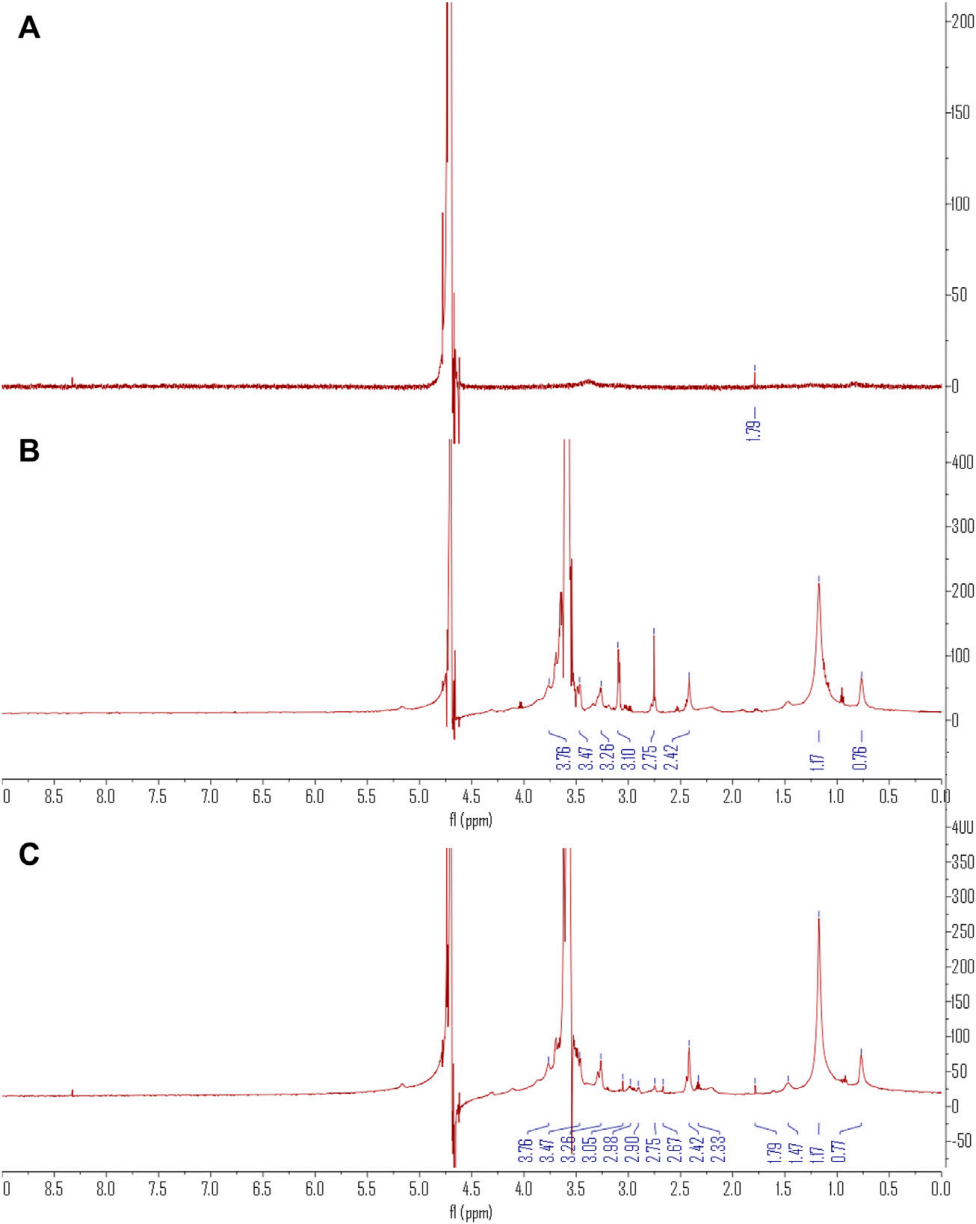
The ^1^H NMR spectra of C60(OH)_22_ **(A)**, DSPE-PEG-NH_2_ **(B)**, and DSPE-PEG-C60 **(C)**.

The ^1^H NMR spectra in Figure 2 show the combination of C60(OH)_22_ and DSPE-PEG-NH_2_ in the DSPE-PEG-C60 spectrum. A single peak at δ of 1.79 representing the proton of C=C-C-H in C60(OH)_22_ and the PEG region of DSPE-PEG at δ of 3.47–3.76 was observed. Peaks at δ of 3.26 ppm (d), δ3.05 ppm (s), δ2.98 ppm (m), δ2.90 ppm (d), δ2.75 ppm (m), δ2.67 ppm (s), δ2.42 ppm (d), δ2.33 ppm (m), δ1.47 ppm (s), δ1.17 ppm (s), and δ0.77 ppm (s) also showed characteristics of DSPE-PEG.

**FIGURE 3:**
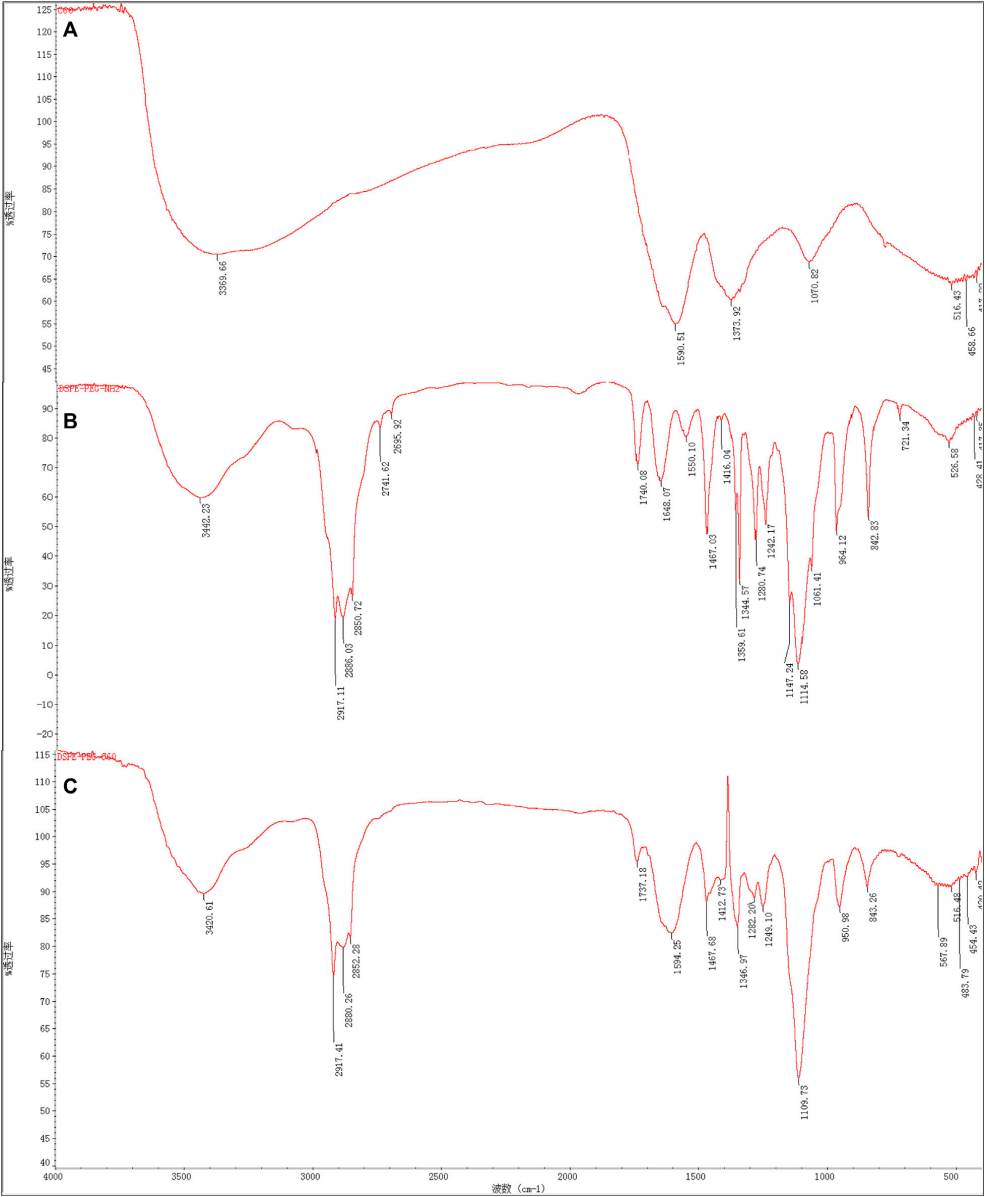
The FT-IR spectra of C60(OH)_22_ **(A)**, DSPE-PEG-NH_2_ **(B)**, and DSPE-PEG-C60 **(C)**.

From the FT-IR spectrum of Figure 3, the conjugation of C60(OH)_22_ to DSPE-PEG-NH_2_ is confirmed. Peaks of DSPE-PEG-C60 (Figure 3C) at 3,421; 2,917; 2,880; 2,852; 1,737; 1,594; 1,468; 1,413;1,347; 1,282; 1,249; 1,110; 951; and 843 (cm^−1^) indicated the absence of the N-H bending vibration of the primary amine peak of DSPE-PEG-NH_2_ at 1,648 and 1,550 cm^−1^ (Figure 3B), and the existence of the -C= C- groups of C60(OH)_22_ with a broad peak at 1,594 cm^−1^ indicated the successful coupling of the carbon–carbon double bond of C60(OH)_22_ with the amine group of DSPE-PEG-NH_2_.

**FIGURE 4:**
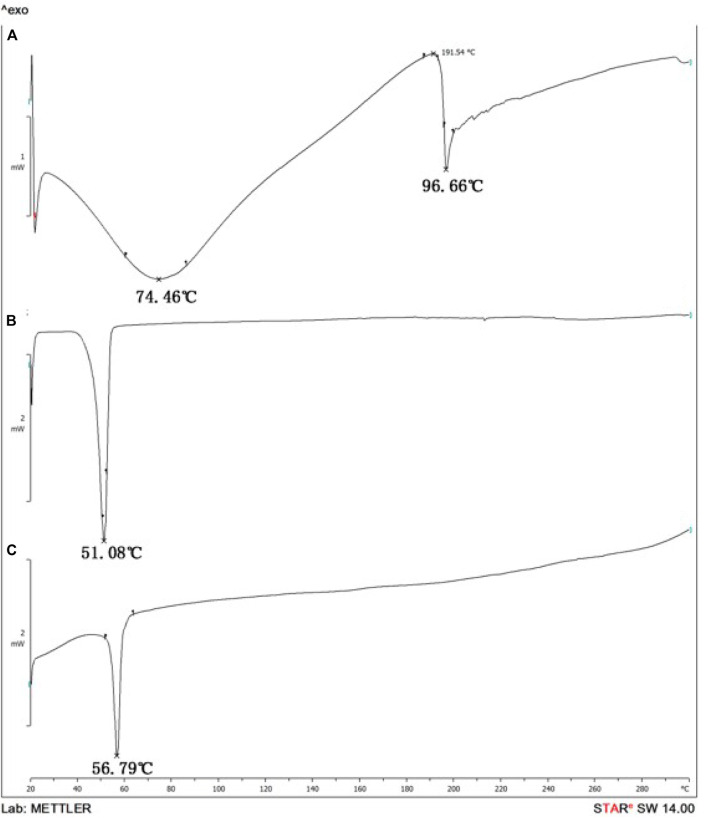
Differential Scanning Calorimetry (DSC) spectrum: C60(OH)_22_ **(A)**, DSPE-PEG-NH_2_ **(B)**, and DSPE-PEG-C60 **(C)**.

When compared to the spectra of the raw materials, the product DSPE-PEG-C60 (Figure 4C) has a sharp endothermic peak at 56.79°C, different from DSPE-PEG-NH_2_ (Figure 4B) and C60(OH)_22_ (Figure 4A), which have a sharp peak at 51.08°C and a large peak at 74.46°C. The DSC spectrum shows that the product is not the same substance or is a mixture of raw materials.

### 3.2 Preparation and Characterization of Micelles

**FIGURE 5:**
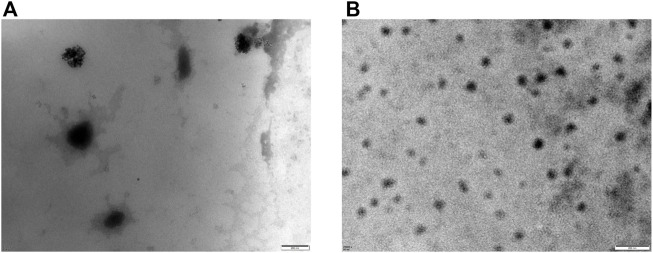
Transmission electron microscopy (TEM) images of micelles: DSPE-PEG-C60: DOX = 10:1 (W/W) **(A)** and DSPE-PEG-NH_2_: DOX = 10:1 (W/W) **(B)**.

TEM images of the micelles [vector: DOX = 10:1(W/W)] are shown in Figure 5. Compared to micelles with DSPE-PEG-NH_2_ as the carrier, micelles prepared with DSPE-PEG-C60 were larger in diameter due to the introduction of C60(OH)_22_. DLS indicated that the mean diameters of the DSPE-PEG-C60 micelles were approximately 97 nm [DSPE-PEG-C60: DOX = 15:1(W/W)], 211 nm [DSPE-PEG-C60: DOX = 10:1 (W/W)], and 260 nm [DSPE-PEG-C60: DOX = 5:1(W/W)], respectively. The diameters of the DSPE-PEG micelles ranged from 10 to 23 nm, which were much smaller than those of the former version (Table 1).

**TABLE 1:** Particle size distribution (PSD), PDI, zeta potential (ZP), encapsulation efficiency (EE), and drug loading content (LC) of micelles.

	DSPE-PEG-C60: DOX = 5:1 (W/W)	DSPE-PEG-C60: DOX = 10:1 (W/W)	DSPE-PEG-C60: DOX = 15:1 (W/W)	DSPE-PEG-NH_2_: DOX = 5:1 (W/W)	DSPE-PEG-NH_2_: DOX = 10:1 (W/W)	DSPE-PEG-NH_2_: DOX = 15:1 (W/W)
PSD (nm)	259.90 ± 11.41	211.30 ± 16.68	96.50 ± 11.39	23.40 ± 0.97	11.83 ± 0.06	9.73 ± 0.09
PDI	0.321 ± 0.024	0.306 ± 0.008	0.322 ± 0.078	0.335 ± 0.007	0.323 ± 0.002	0.315 ± 0.044
ZP (mv)	−28.67 ± 1.68	−29.53 ± 1.22	−30.87 ± 0.25	+7.80 ± 0.64	+6.93 ± 032	+6.47 ± 0.10
EE (%)	86.1 ± 1.4	95.4 ± 0.6	97.5 ± 0.4	97.2 ± 0.5	98.0 ± 0.2	98.3 ± 0.2
LC (%)	14.2 ± 0.28	8.6 ± 0.08	6.1 ± 0.04	16.1 ± 0.08	8.9 ± 0.01	6.1 ± 0.01

The DSPE-PEG-C60 micelles have zeta potentials ranging from −30.87 to −28.67 mV with a negative charge and a comparatively high encapsulation efficiency of 86.1, 95.4, and 97.5% when the weight ratio of DSPE-PEG-C60: DOX is more than 5:1. Meanwhile, the zeta potential of the DSPE-PEG micelles ranged from +6 to +8 mV with a positive charge and a high encapsulation efficiency of approximately 98%.

### 3.3 Drug Release Study of Micelles

**FIGURE 6:**
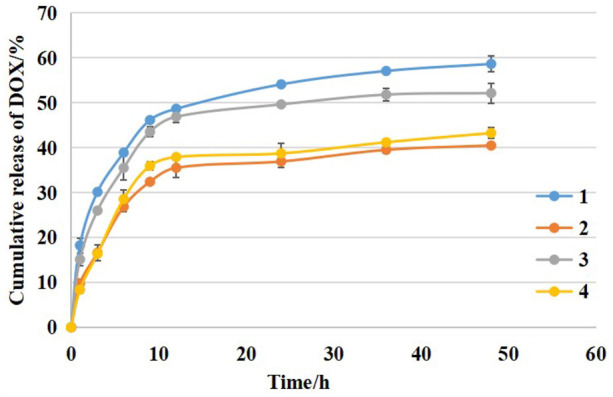
The cumulative release kinetics of DOX from micelles: DSPE-PEG-C60: DOX = 10:1 (W/W) **(1)**, DSPE-PEG-C60: DOX = 15:1 (W/W) **(2)**, DSPE-PEG-NH_2_: DOX = 10:1 (W/W) **(3)**, and DSPE-PEG-NH_2_: DOX = 15:1 (W/W) **(4)** in PBS buffer (pH 7.4) at 37°C.

The stabilities of the micelles were examined by evaluating the cumulative release of DOX in phosphate buffer (pH 7.4). As shown in Figure 6, after 48 h of incubation, the cumulative release rates of DOX from the DSPE-PEG-C60 micelles were found to be 57% [(DSPE-PEG-C60: DOX = 10:1(W/W)] and 41% [(DSPE-PEG-C60: DOX = 15:1(W/W)], respectively, while those from DSPE-PEG micelles were 52% [(DSPE-PEG-NH_2_: DOX = 10:1(W/W)] and 42% [(DSPE-PEG-NH_2_: DOX = 15:1(W/W)], respectively. These results indicated that the introduction of C60(OH)_22_ did not significantly alter the release kinetics of the micelles.

### 3.4 *In vitro* Cytotoxicity Examination

**FIGURE 7:**
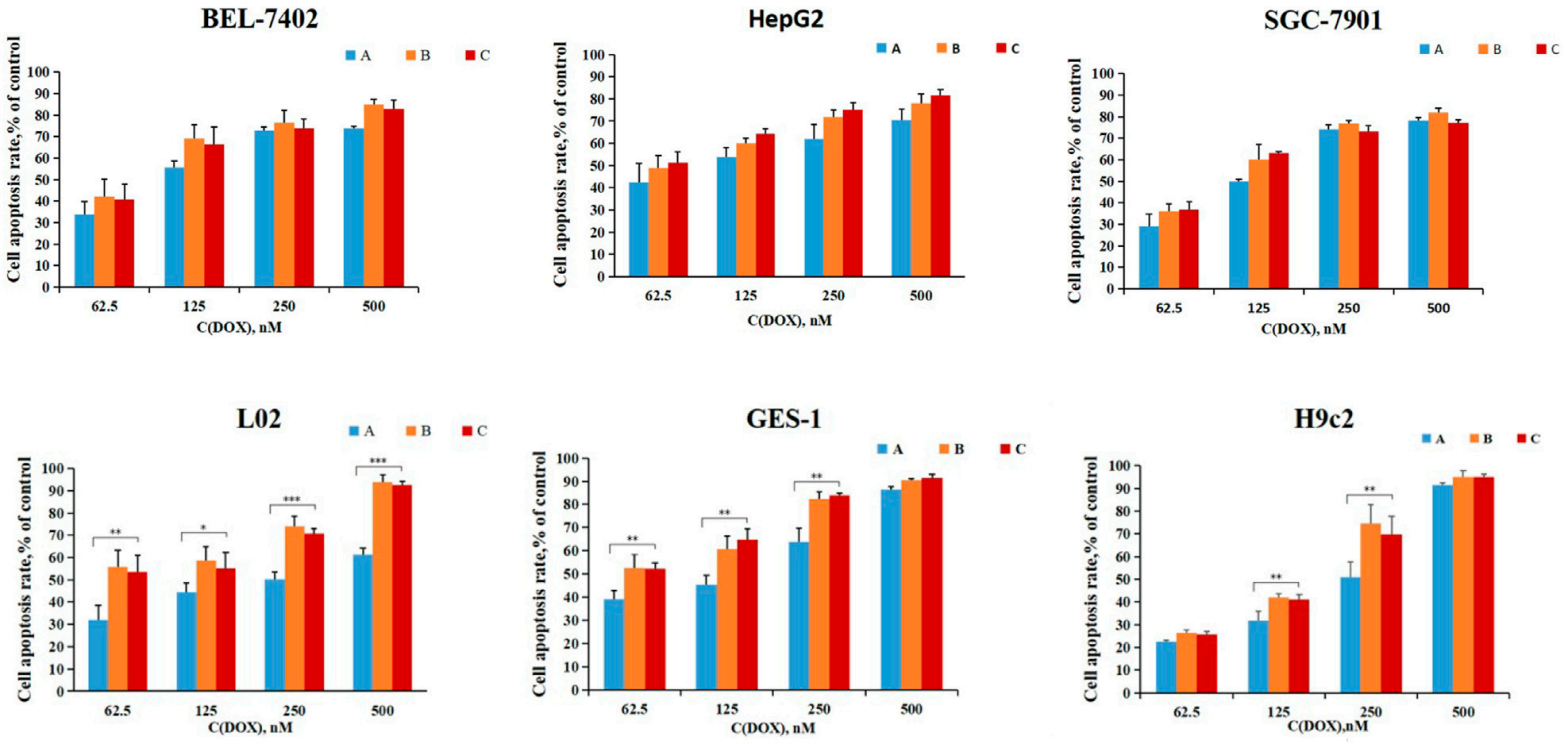
*In vitro* cytotoxicity of micelles: DSPE-PEG-C60: DOX = 10:1 (W/W) (A), DSPE-PEG-NH_2_: DOX = 10:1 (W/W) (B), and free DOX (C) for 72 h for the determination of cell death using CCK-8 assays. Data represent the mean ± SD from three independent experiments (*p < 0.05, **p < 0.01 and ***p < 0.001).

The cytotoxicity of the micelles [DSPE-PEG-C60: DOX = 10:1 (W/W)], [DSPE-PEG-NH_2_: DOX = 10:1 (W/W)] was compared to free DOX on the HepG2, BEL-7402, SGC-7901, GES-1, L02, and H9c2 cell lines. As shown in Figure 7, there were no significant differences in the cell apoptosis rate between DSPE-PEG-C60 micelles, DSPE-PEG micelles, and DOX against carcinoma cell lines, such as HepG2, BEL-7402, and SGC-7901, which indicated that the prepared DSPE-PEG-C60 micelles loaded with DOX had little influence on tumor inhibition *in vitro*.

However, DSPE-PEG-C60 micelles showed significantly weaker cytotoxicities against normal cells (L02, GES-1, and H9c2) than DSPE-PEG micelles and free DOX, especially in the L02 case. The apoptosis rate of the cells treated with DSPE-PEG-C60 micelles was approximately 30% less than that of the cells treated with free DOX at a concentration of 500 nM in the L02 cell line and approximately 20% less than that of the positive control at a concentration of 250 nM in the H9c2 and GES-1-cell lines. This result suggested that the introduction of C60(OH)_22_ in the form of micelles could reduce the cytotoxicity induced by DOX against normal cell lines.

### 3.5 Cell Apoptosis Detection

**FIGURE 8:**
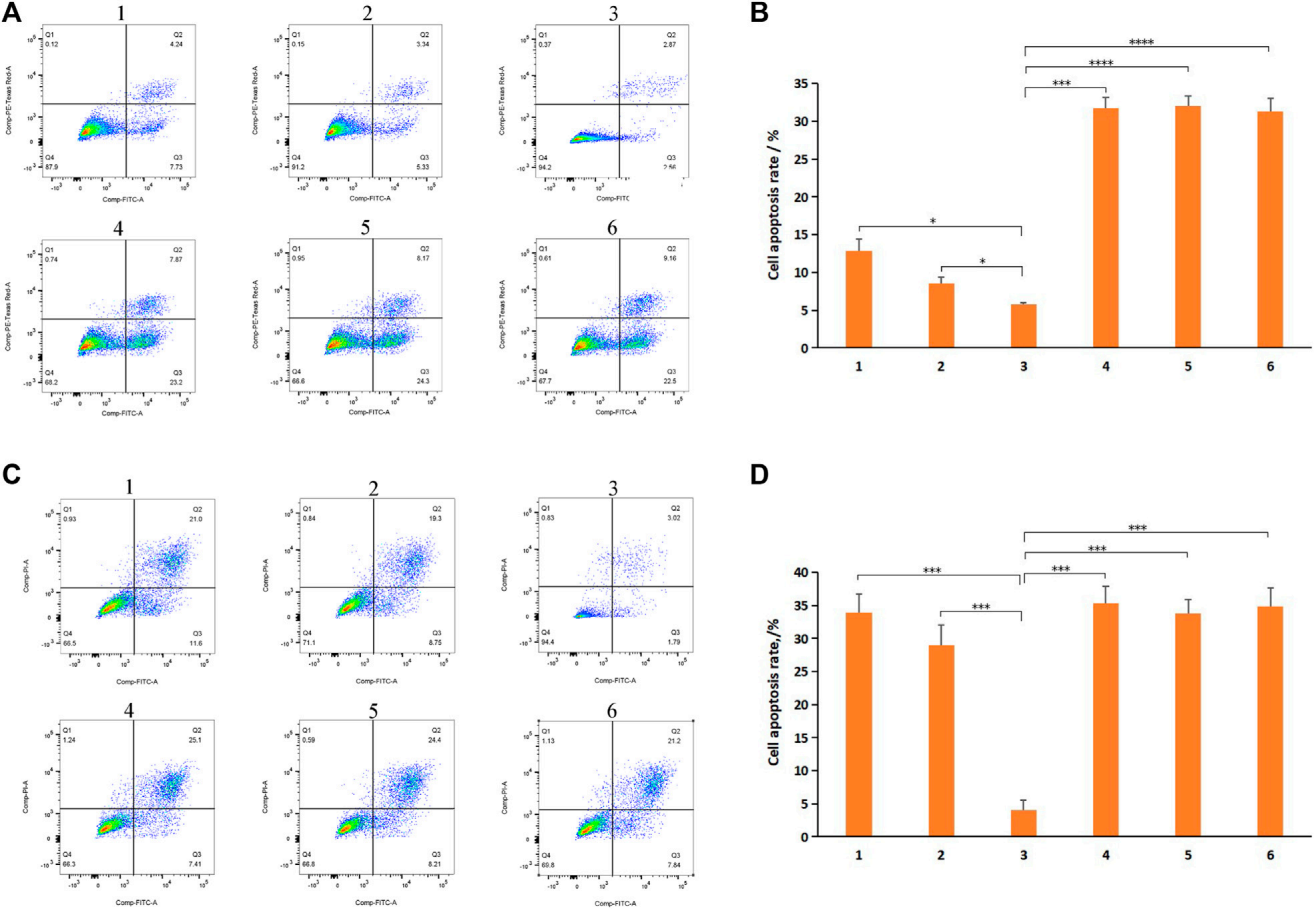
Effects of micelles and free DOX on apoptosis of H9c2 cells and BEL-7402 cells: DSPE-PEG-C60: DOX = 10:1 (W/W) (1), DSPE-PEG-C60: DOX = 15:1 (W/W) (2), control (3), DSPE-PEG-NH_2_: DOX = 10:1 (W/W) (4), DSPE-PEG-NH_2_: DOX = 15:1 (W/W) (5), and free DOX (6). Cells were treated with micelles or free DOX at equivalent concentrations of 500 nM DOX (H9c2 cells) or 1,000 nM DOX (BEL-7402 cells) for 24 h, followed by annexin V/PI staining and flow cytometry detection. **(A)** Representative pseudo-color plots of annexin V/PI staining of H9c2 cells. **(B)** Apoptosis ratio of the calculated H9c2 cells. **(C)** Representative pseudo-color plots of Annexin V/PI staining of BEL-7402 cells. **(D)** Apoptosis ratio of the calculated BEL-7402 cells. The values presented are the means ± standard deviations of three independent experiments (*p < 0.03, ***p < 0.001, ****p < 0.0001).

To examine whether the observed decreased cytotoxicity against normal cell lines was the result of the inhibition of apoptosis by DSPE-PEG-C60 micelles, H9c2 cells were subjected to apoptosis detection. Additionally, micelles composed of [DSPE-PEG-C60: DOX = 10:1 (W/W)] and micelles composed of [DSPE-PEG-C60: DOX = 15:1 (W/W)] were used to estimate the necessity of the increase in C60(OH)_22_ in the delivery system. Annexin V-FITC/PI double staining with flow cytometry was used. The representative pseudo-color plots (Figure 8A) and the apoptosis ratio of the cells (Figure 8B) indicated that doxorubicin treatment induced a high apoptosis rate, up to 31%, at a DOX concentration of 500 nM. DSPE-PEG-C60 micelles inhibited the toxicity induced by DOX with a decreased apoptosis ratio of 12.9% [DSPE-PEG-C60:DOX = 10:1 (W/W)] and 8.6% [DSPE-PEG-C60: DOX = 15:1 (W/W)] compared to the 5.8% apoptosis rate of the control group. These results indicate a correlation between the dose of C60(OH)_22_ and the protection against doxorubicin-induced cytotoxicity.

However, it would not be good if the effect of C60(OH)_22_-modified DOX-loaded micelles against tumor cells reduced. BEL-7402 cells were subjected to apoptosis detection to examine whether the cytotoxicity of DSPE-PEG-C60/DOX micelles against tumor cells was significantly reduced. The representative pseudo-color plots (Figure 8C) and the apoptosis ratio of the cells (Figure 8D) indicated that DSPE-PEG-C60 micelles [vector: DOX = 15:1(W/W)] had a slightly decreased apoptosis ratio of approximately 29%, while DSPE-PEG-C60 micelles [vector: DOX = 10:1(W/W)] induced an approximately 34% apoptosis rate at 1,000 nM DOX concentration without a significant difference compared to the free DOX group or other micelle groups.

### 3.6 Intracellular ROS Level Evaluation

**FIGURE 9:**
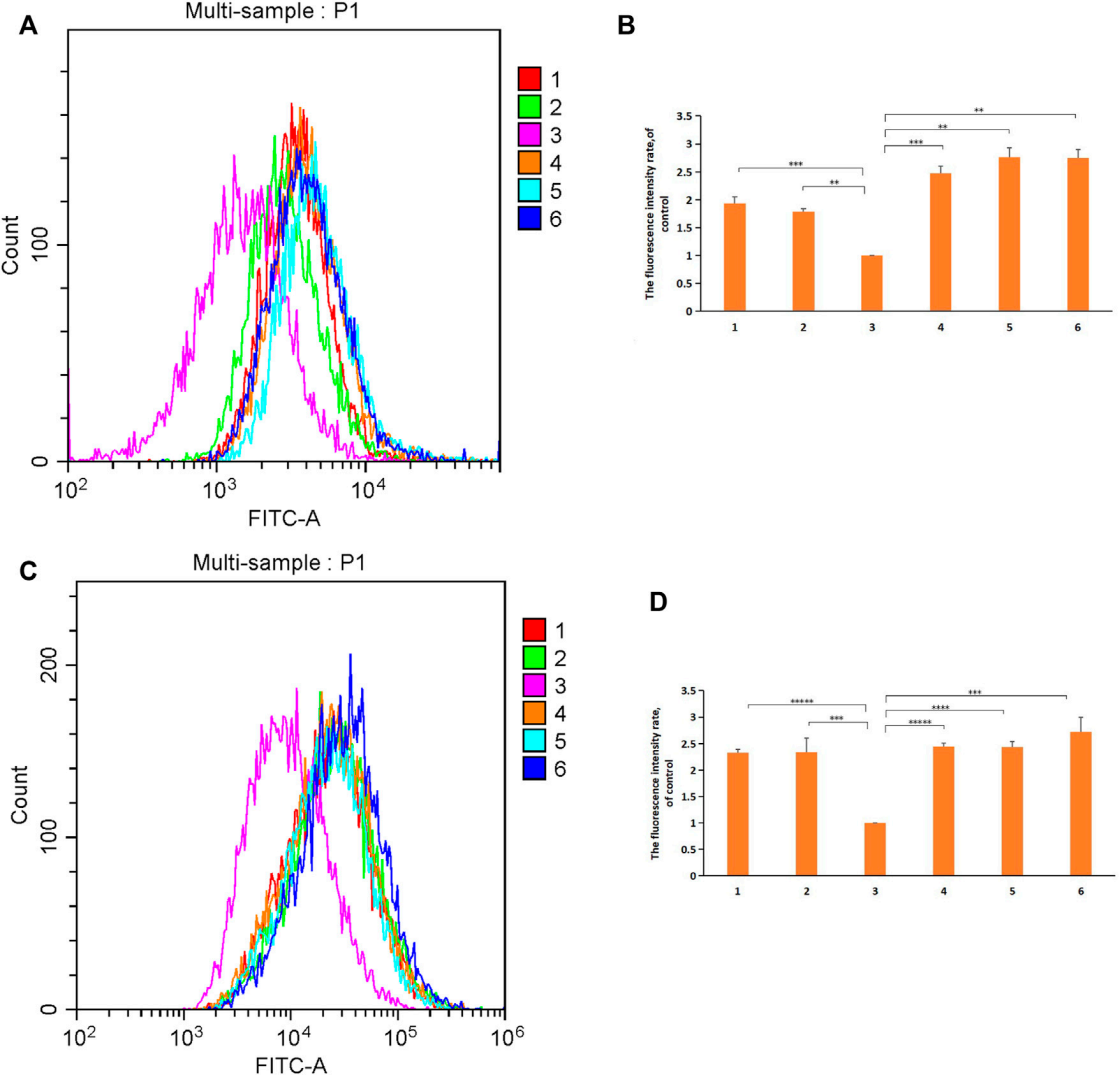
Effect of micelles and free DOX on ROS levels in H9c2 cells and BEL-7402 cells: DSPE-PEG-C60: DOX = 10:1 (W/W) (1), DSPE-PEG-C60: DOX = 15:1 (W/W) (2), control (3), DSPE-PEG-NH_2_: DOX = 10:1 (W/W) (4), DSPE-PEG-NH_2_: DOX = 15:1 (W/W) (5), and free DOX (6). Cells were treated with micelles or free DOX for 24 h, followed by DCFDA staining and flow cytometry detection. **(A)** Representative fluorescence intensity plots of DCF in H9c2 cells. **(B)** Fluorescence intensity rate of the calculated H9c2 cells. **(C)** Representative fluorescence intensity plots of DCF in BEL-7402 cells. **(D)** Fluorescence intensity rate of the calculated BEL-7402 cells. The values presented are the means±standard deviations of three independent experiments (**p < 0.01, ***p < 0.001, ****p < 0.0001, and *****p < 0.00001).

Differences in Reactive Oxygen Species (ROS) levels in H9c2 cell lines treated with micelles or free DOX were assessed using DCFDA. Free DOX-induced oxidative stress in H9c2 cells (Moloney and Cotter, 2018) resulted in a threefold increase in DCF fluorescence compared to untreated cells. DSPE-PEG-C60 micelles had a significantly lower DCF level compared to the DSPE-PEG micelle groups and the free DOX group (Figures 9A,B), whose DCF levels were approximately 30% less [DSPE-PEG-C60: DOX = 10:1(W/W)] and 35% less [DSPE-PEG-C60: DOX = 15:1(W/W)] than the group treated with free DOX. On the other hand, treatment with DSPE-PEG micelles showed no differences versus the free DOX group. These results indicated the strong capability of C60(OH)_22_ to attenuate oxidative stress in H9c2 cells.

ROS levels in BEL-7402 cells treated with micelles or free DOX were also assessed (Figures 9C,D). The DCF levels of the DSPE-PEG-C60 micelle groups were not significantly different from those of the free DOX group or the DSPE-PEG micelle groups. Meanwhile, an approximately threefold increase in ROS production was detected in untreated BEL-7402 cells compared with untreated H9c2 cells, which led to the higher ROS levels in the BEL-7402 cells than the H9c2 cells. The reduced influence of the antioxidative stress activity of C60(OH)_22_ on BEL-7402 cells with baseline high ROS levels is an important reason for the unimproved viability of the tumor cells treated with DSPE-PEG-C60 micelles.

### 3.7 Cell Mitochondrial Function Evaluation

**FIGURE 10:**
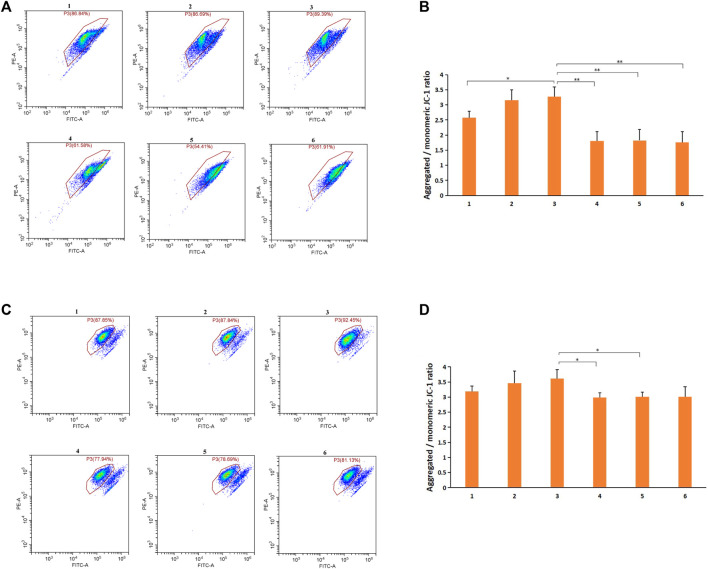
Effects of micelles and free DOX on the mitochondrial membrane potential of H9c2 cells and BEL-7402 cells: DSPE-PEG-C60: DOX = 10:1 (W/W) (1), DSPE-PEG-C60: DOX = 15:1 (W/W) (2), control (3), DSPE-PEG-NH_2_: DOX = 10:1 (W/W) (4), DSPE-PEG-NH_2_: DOX·HCl = 15:1 (W/W) (5), and free DOX (6). Cells were treated with micelles or free DOX·HCl for 24 h. Mitochondrial membrane potential (ΔψM) was measured by JC-1 staining, followed by flow cytometry. **(A)** Representative pseudo-color plots of JC-1 staining of H9c2 cells. **(B)** Graphs represent the ratio of red fluorescence (JC-1 aggregates) to green fluorescence (JC-1 monomers) compared to the control H9c2 cells. **(C)** Representative pseudo-color plots of JC-1 staining of BEL-7402 cells. **(D)** Graphs represent the ratio of red fluorescence (JC-1 aggregates) to green fluorescence (JC-1 monomers) compared to the control BEL-7402 cells. Data are represented the means±standard deviations from three independent experiments (*p < 0.05, **p < 0.01).

To investigate whether the functional integrity of mitochondria was preserved after treatment with DSPE-PEG-C60 micelles, JC-1 staining was performed. H9c2 cells were treated with 500 nM free DOX or micelles loaded with DOX as described previously. After JC-1 loading, the cells were immediately detected by flow cytometry. Healthy cells with high mitochondrial membrane potential (ΔψM) formed JC-1 aggregates showing red fluorescence, while apoptotic cells with low ΔψM exhibited green fluorescence. Treatments with DSPE-PEG-C60 micelles had a relatively high ratio of red fluorescence (JC-1 aggregates) to green fluorescence (JC-1 monomers) of 2.6-fold [DSPE-PEG-C60: DOX = 10:1 (W/W)] and 3.2-fold [DSPE-PEG-C60: DOX = 15:1 (W/W)], compared to the 3.4-fold ratio of the control group. In contrast, both DOX treatment and DSPE-PEG micelle treatments caused a decrease in JC-1 aggregates with a reduced red-to-green fluorescence ratio of approximately 1.8-fold compared to the 3.4-fold ratio in untreated cells, indicating serious mitochondrial damage in H9c2 cells (Figures 10A,B).

Then, BEL-7402 cells were treated with 1,000 nM free DOX or micelles loaded with DOX and examined as described previously. The results indicated that the red-to-green fluorescence ratio of the DSPE-PEG-C60 micelle groups was not significantly different from that of the free DOX group or the DSPE-PEG micelle groups, although the former showed a slight increase in the normal cell population (Figures 10C,D).

## 4 Discussion

Nanoparticles are widely used in antitumor drug delivery systems due to their passive targeting ability in tumor tissue, which has an enhanced permeation and retention effect (EPR effect). However, the Warren C.W. Chan team presented in 2020 that the exposure of nanomedicines in tumor tissue was only 0.7% of the intravenous dose, much less than the 40% of the total administration dose of the earlier estimates (Sindhwani et al., 2020; de Lázaro and Mooney, 2020; Pandit et al., 2020). Although the result of the former is still debated, it poses serious challenges for the design of antitumor drug delivery systems (DDSs) in the future, and DDSs with reduced toxicity to normal tissues, as well as improvements in delivery efficiency, have advantages over traditional DDSs.

Nanopharmacotherapeutics with particle sizes ranging from 100 to 200 nm show higher tumor tissue accumulation than nanoparticles with particle sizes greater than 300 nm or less than 50 nm (Seymour et al., 1995; Noguchi et al., 1998; Maeda et al., 2001). Most micelles are typically approximately 10–50 nm in size, such as the DSPE-PEG micelles in this study, which ranged from 10 to 23 nm. Thus, the DSPE-PEG-C60 micelles were more suitable for the EPR effect with a size of 96.5 nm [(DSPE-PEG-C60: DOX = 15:1(W/W)] and 211.3 nm [(DSPE-PEG-C60: DOX = 10:1(W/W)] than the DSPE-PEG micelles.

Polymeric materials with cationic surfaces easily interact with negatively charged vascular endothelial cells, cytoplasm, or plasma components, shortening their half-lives and activating intracellular complement in T cells, leading to cell toxicity (Ilinskaya, et al., 2019). DSPE-PEG micelles could be unstable in the bloodstream due to their positive charges. In contrast, DSPE-PEG-C60 micelles with zeta potentials ranging from −30.87 to −28.67 mV are expected to have better stability and a longer life than DSPE-PEG micelles *in vivo*.

Finally, fullerenols have long been studied for their role in organ protection against DOX in rats. The cell apoptosis effects of DSPE-PEG-C60 micelles and DSPE-PEG micelles were studied in tumor and normal cell lines. DSPE-PEG-C60 micelles showed great protectiveness against DOX for the normal cell lines in this study, with a maximum decrease of approximately 20–30% in the apoptosis ratio on normal cell lines (L02, GES-1, and H9c2), compared to that of the control group. Interestingly, in experiments with tumor cell lines, the introduction of C60(OH)_22_ to the micelles did not seem to significantly affect the final tumor suppressive effect. This suggests that the inhibitory effect of DSPE-PEG-C60 micelles on cells is related to the type of cells, which may be due to the difference in the structural features and metabolism of tumor cell lines from normal cell lines, such as the high level of ROS detected in various cancer cells (Moloney and Cotter, 2018).

## 5 Conclusion

In this study, we prepared DSPE-PEG-C60 micelles loaded with DOX for treating human cancers. DSPE-PEG-C60 micelles have sizes ranging from 90 to 250 nm and a negative zeta potential, which exhibited an obvious decrement of cytotoxicity toward normal cell lines and unchanged antitumor efficacy against tumor cell lines. The effect of the DSPE-PEG-C60 micelles toward DOX-induced cardiotoxicity in cardiomyocytes was then studied. We demonstrated that DSPE-PEG-C60 micelles protected H9c2 cells from doxorubicin by reducing excessive intracellular ROS production, restoring the mitochondrial membrane potential, and modulating apoptosis function. Further investigations are in progress to examine their safety in clinically relevant animal models.

Overall, this study provided a fullerenol-modified micellar system that is friendly to normal cells, which has the potential to be used as a drug carrier in cancer treatment.

## Data Availability

The raw data supporting the conclusions of this article will be made available by the authors, without undue reservation.
